# The use of unmanned aerial vehicles for health purposes: a systematic review of experimental studies

**DOI:** 10.1017/gheg.2018.11

**Published:** 2018-06-27

**Authors:** R. M. Carrillo-Larco, M. Moscoso-Porras, A. Taype-Rondan, A. Ruiz-Alejos, A. Bernabe-Ortiz

**Affiliations:** 1 CRONICAS Center of Excellence in Chronic Diseases, Universidad Peruana Cayetano Heredia, Lima, Peru; 2 Department of Epidemiology and Biostatistics, School of Public Health, Imperial College London, London, UK; 3 Faculty of Health Sciences, Universidad Peruana de Ciencias Aplicadas – UPC, Lima, Peru; 4 Faculty of Epidemiology and Population Health, London School of Hygiene and Tropical Medicine, London, UK

**Keywords:** Disasters, drones, emergencies, healthcare, unmanned aerial vehicles

## Abstract

**Background:**

Unmanned aircraft vehicles (UAVs) have had a rapid escalation in manageability and affordability, which can be exploited in healthcare. We conducted a systematic review examining the use of drones for health-related purposes.

**Methods:**

A search was conducted in Medline, Embase, Global Health, Scopus, CINAHL and SciELO. Experimental studies were selected if the population included human subjects, the intervention was the use of UAVs and there was a health-related outcome.

**Results:**

Of 500 results, five met inclusion criteria during an initial search. An updated search yielded four additional studies. Nine studies, all in high-income countries, were included for systematic syntheses: four studies addressed out-of-hospital cardiac arrest emergencies, three assessed drones for identification of people after accidents, one used drones to transport blood samples and one used drones to improve surgical procedures in war zones.

**Conclusions:**

Research on the use of drones in healthcare is limited to simulation scenarios, and this review did not retrieve any studies from low- and middle-income countries.

## Background

Different technologies have been evaluated as potential means to improve healthcare [1, 2]. For example, mobile health, or mHealth, has used mobile phones and short message services (SMS) with positive effects in several fields such as sexual health [3], child and maternal health [4] and chronic conditions [5, 6]. Furthermore, reviews on the use of technology for patient monitoring and diagnostics, such as Telemedicine, suggest that they can be both acceptable for patients and cost-effective in healthcare delivery [7, 8]. Although further studies are needed in order to improve the quality of scientific evidence on the use of these technologies [2], there is also a need to explore other technology-based solutions for situations and scenarios where healthcare needs surpass the applicability and functionality of mobile phones. In this line, unmanned aircraft vehicles (UAVs), including drones, can be used for various health purposes, from finding survivors after natural disasters to delivering medicines or providing care in emergency situations. Unlike the increasing body of evidence on the use of mobile phones for health purposes [2–6], to the best of our knowledge, no systematic review of the use of UAVs for health-related purposes has been published. Consequently, we intended to close this knowledge gap by conducting a systematic review to assess the potential usability of UAVs in healthcare. We aimed to assess experimental studies in which UAVs were used for health-related purposes, particularly in emergency situations. Thus, we sought to answer the research question: regarding health-related outcomes, what has been the use of drones?

## Methods

### Study design

This is a systematic review of the literature. PRISMA guidelines were followed (Online Supplementary Material 1) [9, 10], and the study was registered in PROSPERO (CRD42017072194).

### Search

Studies were selected for this review if their population was men or women of any age, the intervention used UAVs and if they assessed any health-related outcome (e.g. survival or time to rescue injured patients). Because, to the best of our knowledge, this would be the first review about this topic, we did not target any specific comparison for the intervention. We only focused on experimental studies, either those conducted in real-life scenarios or simulation studies. Experimental studies (e.g. clinical trials) provide the strongest evidence to support the use of an intervention or treatment. Therefore, in an attempt to summarize the most robust evidence about the use of drones in health-related outcomes, we focused on this design alone.

The search was conducted in OVID including Embase (1974–2017), Global Health (1910–2017) and MEDLINE (1946–2017); we also searched in SciELO, Scopus and CINAHL. All database searches were conducted on 1 June 2017, except Scopus, which was conducted on 6 June. Following the initial search, new relevant articles were found on social media, so an updated search was conducted on 14 October (Ovid and Scopus) and 17 October (SciELO and CINAHL), 2017. No grey literature was searched.

Table 1 depicts the terms used for the search, which included drone-related and health outcomes terms. Drone-related terms were verified by an engineer with experience in drones. Online Supplementary Material 1 depicts the search conducted in OVID, CINAHL and Scopus.

**Table 1.** Search terms

Drone-related terms	Unmanned aerial vehicle
	UAV
	Unmanned aircraft system
	UAS
	Unmanned aerial system
	Drone
	Quadcopter
Health-related outcomes terms	Mortality
	Mortality, premature
	Treatment outcome
	Rescue work
	Disasters
	Disasters victims
	Disaster medicine
	Disaster planning
	Morbidity
	Emergencies
	Emergency responders
	Emergency treatment
	Emergency medicine
	Emergency medical services
	Paediatric emergency medicine

### Study selection

Results from each search engine were downloaded and duplicates were excluded. Then, all the results were divided in two groups, each of which was studied in terms of titles and abstracts by two researchers independently (RMC-L and AT-R; MM-P and AR-A). Discrepancies between the reviewers were resolved by a third reviewer (AB-O). After initial review, full text of selected studies was independently assessed by two reviewers (MM-P and AT-R). Again, discrepancies were resolved by a third reviewer (AB-O). Additional findings of the updated search were reviewed by one researcher (RMC-L) and verified by another one (AB-O).

### Data extraction

Data extraction from each selected study was conducted by two reviewers independently (RMC-L and AR-A), and later revised by a third one (AB-O). Before information synthesis, a data extraction form was developed by the reviewers based on the research question (e.g. study design, intervention allocation and effect of the intervention); the data extraction form was not modified during information synthesis and all the information is depicted in Table 2. No further validation process of the data extraction form was conducted. Because all relevant information was available on the research papers, it was not necessary to request further details from the investigators of each selected study. To the best of our knowledge, no specific tools have been developed for risk of bias assessment of simulation studies; therefore, we were unable to assess the risk of bias in our review. Because we aimed to summarize the use of UAVs in healthcare, and we had anticipated a great heterogeneity among studies, a meta-analysis was not conducted. Nevertheless, if the selected studies presented any summary measures (e.g. risk ratio), these were summarized.

**Table 2.** Characteristics of the selected studies for systematic synthesis

First author (ref)	Country (year of publication)	Study design	Number of subjects in intervention/control group (if applicable)	Time invested in the development of the device for this experiment (months)/number of devices used in the experiment	What did the device do?	Health-related outcome assessed	Effect of the use of the device
Abrahamsen [11]	Norway (2015) Experiment conducted in 2013	Pilot feasibility study using simulated emergency scenarios	One drone but five simulated scenarios Simulation #1: 25 children simulated to be injured and trapped passengers in a bus Simulation #2: one simulated injured skier Simulation #3: unknown number of skiers after an avalanche Simulation #4: a person simulated that had broken through thin ice Simulation #5: none	One device (drone) per experiment (simulation) guided by specialist	Simulation #1: identified the emergency scenario (e.g. kind of accident, number of vehicles involved and damages); also, identification of victims [number and overall state (detection of respiratory movements)]; infrared camera revealed victims inside a dark bus Simulation #2: video recording of the emergency scenario (similar quality to that one recorded by a manned helicopter); it could get closer to the victim and identify respiratory movements Simulation #3: remote aerial visual search of survivors Simulation #4: used as a tool carrier; transported and dropped tools close to the victim Simulation #5: laser beam used to point objects on the ground, and infrared camera detected body-sized silhouettes and warm objects	Simulation #1: wellbeing and rescuing of simulate injured and trapped passengers Simulation #2: aiding an accident victim where other health staff could not approach Simulation #3: identification of number of victims after a natural disaster Simulation #4: providing tools to assist accident victims Simulation #5: identification and locating lost subjects	Simulation #1: correct identification of number of vehicles involved in the accident, as well as number of victims and his/her states of conscious Simulation #2: correct identification of respiratory movements of a found victim Simulation #3: victims were found and their position correctly identified and informed Simulation #4: victims successfully received tools Simulation #5: victims were correctly identified, through body silhouettes and warm objects
Claesson [18]	Sweden (2017) Experiment conducted in October 2016	Simulation experiment aimed to compare delivery time of an automated external defibrillator by a drone *v.* regular emergency system (no real-time simultaneous comparator)	18 consecutive autonomous remotely operated flights were performed	One device dispatched for flights during a 72-h period to locations where consecutive out-of-hospital cardiac arrests within a 10-km radius from the fire station had occurred between 2006 and 2014	Provide an automated external defibrillator for consecutive out-of-hospital cardiac arrests	Time from dispatch to arrival of the drone at the scene of the out-of-hospital cardiac arrest compared with time for emergency medical services	Reduced time to provide automated external defibrillator to patient with cardiac arrest compared with regular emergency system: time median reduction of 16:39 (95% CI 13:48–20:12, *p* < 0.001) min
Claesson [12]	Sweden (2016) Experiment conducted in (not specified)	Explorative study to describe the potential benefit and the practical use of a drone system to decrease response time in out-of-hospital cardiac arrest using theoretical modelling and simulation	3165 out-of-hospital cardiac arrests cases (3041 in 10 urban locations and 124 in 10 rural locations) were included in the theoretical GIS model	Two devices operated by two licensed pilots	Provided an automated external defibrillator	Suitable placements and response times for the use of an automated external defibrillator equipped drone	Using simulation models, the drone arrived before the emergency system in 32% of cases (mean time saved with the drone was 1.5 min); in rural areas, the drone arrived before the emergency system in 93% of the cases (mean time saved was 19 min). The latch-release of the automated external defibrillator from low altitude (3–4 m) or lading the drone on flat ground were the safest ways to deliver the defibrillator (superior to parachute release)
Harnett [13]	USA (2008) Experiment conducted in (not specified)	Experimental pilot study to develop and validate UAV-based communication and mobile robotic surgical system that would allow a remote surgeon to effectively operate on an injured soldier regardless his/her location or environment	One test of their principal aim	One device used during 1 week with a mobile surgical robotic system used by two surgeons	Amplify a wireless network to improve access to robotic surgical system thought for war zones	In proposed experiments, surgeons performed several simple surgical tasks such as suturing	The drone could be adapted as a communication platform allowing network connectivity to a robotic surgical device. Surgeons were able to simulate various manoeuvres that surgeons normally perform. Suturing was difficult as the control of the robot required additional refinement. The latency of 200 ms was noticeable by surgeons but it did not interfere with robot control
Mardell [14]	No specified (2014) Experiment conducted in (not specified)	Pilot experimental study aimed to test two different kinds of image transmission send by a drone in a hypothetical case of looking for someone lost in the wilderness	The experiment involved 18 (two female and 16 males) volunteer participants drawn from the general student and research population of a university	One device in six distinct ground images, from mostly open through to heavily forested areas and including some man-made features, were tested. Each ground image sequence contained three simulated rescue targets (isolated person or two/three people in a tight group) giving a total of 18 targets	Target identification for emergency/rescue situations: the captured live images of an area in which a person has been lost	Recue subjects lost in the wilderness according to two methods of target recognition	Superiority of serial visual presentation mode (SVP) of still images over the video-like moving modes, at a wide range of speeds
Pulver [15]	USA (2016) Experiment conducted in (not specified)	Simulation study aimed to identify appropriate location for drones with automated external defibrillator so that they would reach a cardiac arrest emergency faster than the regular emergency system	None	None	Provided an automated external defibrillator in three scenarios: using emergency medical services stations as potential drone launch sites, using only new locations as potential drone launch sites, and using a combination of new locations and emergency medical services as potential drone launch sites	Time response and coverage of cardiac arrest events in out-patient settings	The emergency medical system only reached 4.3% of the cardiac arrests in 1 min, and 96.4% of the demand can be reached within 5 min using current system and facility locations. Using existing stations to launch drones resulted in 80.1% of cardiac arrest demand being reached within 1 min. Allowing new sites to launch drones resulted in 90.3% of the demand being reached in 1 min
Karaka [19]	Turkey (2017) Experiment conducted in (not specified)	Prospective randomized simulation study. The control arm received a classical line search technique, whereas the intervention arm a drone-snowmobile search technique	The scenario consisted of an unconscious victim (same mannequin wearing the same outfits in all experiments) on a snow-covered ground. This scenario was enacted 10 times for each study group	For the intervention group (drone) consisted of three rescuers (one experienced drone pilot, a rescuer monitor, and a certified snowmobile driver) and one brand drone	The drone searched the victim with a camera transmitting real-time images. The scanning began from a height of 40 m, and when an image compatible with a victim was found, the drone descended to improve the transmission. The drone descended to a height of 10 m to inform the exact victim location to the snowmobile rider	Using a simulation model, the study aimed to test if a drone, alongside a snowmobile, improved the process of seeking and locating victims on snow-covered areas. The primary outcome was the comparison between the two study arms regarding contact time with the victim	The drone-based method was able to search a larger area and did so faster (8.9 *v.* 57.3 min, *p* < 0.001)
Amukele [16]	USA (2015) Experiment conducted in (not specified)	Exploratory study to describe the feasibility of transporting blood samples on drones	Two blood samples were withdrawn from 56 volunteers. One set of the paired tubes was flown on drones	One brand drone was used and controlled with a hobbyist radio control link. It was flown above 100 m over the ground, and orbited the flight field within the sight of the pilot	The drone flew with blood samples for between 6 and 38 min	The drones were used to test if drone transportation would have any impact on the quality of biological specimens, particularly on blood samples withdrawn from volunteers for regular chemistry, haematology and coagulations tests	Samples on drones yielded very similar results to those transported terrestrially. Nevertheless, precision was somewhat lower in the samples transported by drones
Boutilier [17]	Canada (2017) Cardiac arrest cases included in the study occurred between 2006 and 2014	Feasibility study to assess if a network of drones could reduce the time an automated external defibrillator reaches a case of out-of-hospital cardiac arrest. The network was designed following a mathematical modelling approach	53 702 out-of-hospital cardiac arrest cases were included	One drone, which maximum forward velocity was 27.8 m/s^2^	The drone had to deliver an automated external defibrillator, in a shorter time lapse compared with the regular emergency system, following the network modelled to optimize where the drone should be located and how many should be hold at that position	A reduction in the time it takes for an out-of-hospital cardiac arrest emergency to receive an automated external defibrillator	Drones did improve the median time an automated external defibrillator takes to arrive to the emergency case. This way, the whole response time for these emergencies was reduced

## Results

### Study selection

**Fig. 1.**
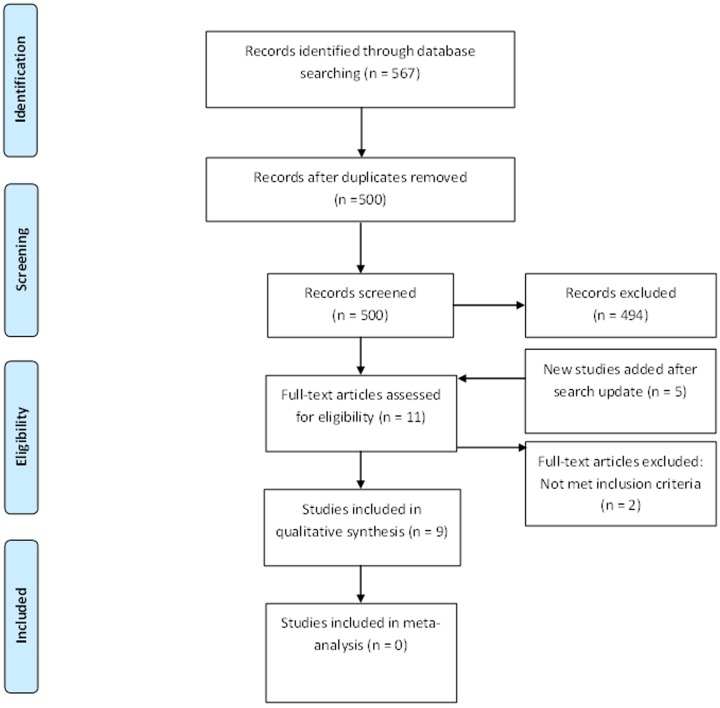
Systematic Search Flow Diagram.

In the initial search, 567 results were retrieved: none from SciELO, 18 from CINAHL, 192 from Ovid and 357 from Scopus. After duplicates were removed, 500 studies were included for initial scrutiny. Titles and abstracts from these 500 studies were examined and six were selected for full-text review. For systematic synthesis, five studies were selected (Fig. 1) [11–15]. The updated search, conducted approximately 4 months afterwards, yielded no new findings from SciELO, but 10 additional titles from CINAHL, 31 from OVID and 29 from Scopus. Four of these new findings were assessed in full-text, and three were included for systematic synthesis [16–18]. Another article was identified after the updated literature search [19]. Overall, after the two search rounds, nine studies (five from the original search, three from the updated search and one additional study) were selected for systematic synthesis and summarized in Table 2. Studies were excluded because they were a narrative review [20] or because they reported an experience using drones to deliver medicines (i.e. not experimental design) [21].

### Study characteristics

All eligible studies were written in the English language, were published since 2008 with three in 2017 and conducted in high-income countries: one in Norway [11], two in Sweden [12, 18], three in the USA [13,15,16], one in Turkey [19], one in Canada [17] and one did not have that information [14]. Only one study had a control group with random allocation of the intervention [19], thus we were unable to compare the studies in terms of study design. None of the studies used drones in real-life situations. All but one [18] were published as original research articles. Further details about the studies’ characteristics are presented in Table 2.

### Use of drones

Eight of the nine studies addressed an emergency situation; one evaluated the use of drones to transport blood samples [16]. In four studies, drones were used in situations of out-of-hospital cardiac arrest (e.g. to transport a defibrillator in a timely manner) [12, 15, 17, 18]; in three reports, drones helped in locating lost or injured people (e.g. in the wilderness or snow-covered ground) [11, 14, 19] and one study used drones to improve wireless connection during surgical procedures in war zones [13]. In all of the studies, the use of drones appears to provide better results than comparison methods. For example, in those addressing out-of-hospital cardiac arrest emergencies, drones improved the time at which a defibrillator was available at the emergency scene and reduced delays in emergency response. Further details about the interventions and outcomes assessed in the selected studies are summarized in Table 2.

## Discussion

### Summary of evidence

This systematic review was conducted using six search engines and found nine reports that studied the use of UAVs for health-related purposes. All the studies were conducted in high-income countries and none were conducted in real-life scenarios. The use of drones included delivering healthcare in out-of-hospital cardiac arrest emergencies, finding lost subjects after accidents or natural disasters, transporting blood samples and as enhancers of other wireless technologies.

The fact that all the studies were in high-income countries could be explained by technological limitations in low- and middle-income countries (LMICs). However, the emergency situations in which drones were helpful aides are not exclusive of high-income countries. In fact, natural disasters often have more devastating impacts in LMICs in terms of deaths and economic loss [22, 23]; suggesting that it may be helpful to test these technologies in LMICs.

Although drones have existed for several years, all the studies found in this systematic review were conducted recently as drones have undergone tremendous technological improvements [24], and a wider use for civil purposes is yet to come [25]. As these technologies become less costly [26], their use in health research will be increasingly affordable.

Most studies followed a simulation methodology, probably under ideal circumstances. However, there is a need to also study drones in real-life situations to identify and overcome potential issues that arise under unfavourable situations, both regarding the health outcome of interest and external factors. Additionally, future studies should take into consideration local regulations for flying drones (e.g. respect of private and public air space), as well as any potential ethical concerns (e.g. respect people's willingness to receive immediate care or wait for further assistance) [27]. Due to the innovative and constantly evolving nature of drone use, ethics committees need to be aware of what permissions or regulations there are to flight drones in the proposed study site, so that they can request and verify that all requirements are met.

### Research and public health implications

Drones have been used in situations where they clearly represent an advantage, like accessing places rescue personnel may not be able to reach [11, 14, 19], or arriving faster when other means of transportation would have limitations [12, 15, 17, 18]. Other potential uses of drones could be to deliver medicines in rural sites with poor connection to cities, to collect environmental information for vector-borne diseases [28] and to measure air pollution [29]. Moreover, drones might be used for delivering pre-hospital medication (e.g. dual antiplatelet therapy) in suspected cases of myocardial infarction where ambulances would take longer than desired. Should drones be used to deliver medicines in rural areas or pre-hospital care in remote areas or where access is difficult, they could help to close health inequalities. Therefore, people who would otherwise face difficulties to access healthcare would have new (and potentially efficient) means to receive care.

### Limitations

Limitations of this systematic review must be acknowledged. First, the instrument used to extract information from the retrieved studies was not validated. This could be a limitation because other important information from these studies could have not been herein presented. Notwithstanding, we believe this instrument extracted what was necessary to answer the research question. In addition, given the few retrieved results, we summarized as much information as possible, and because we did not conduct a meta-analysis or did not retrieve any trial studies, the extracted information shows the overall methodology and results of the selected studies. Furthermore, our findings are less prone to bias because we did not exclude any reports based on the available or extracted information, and all summarized information provide evidence to assess risk of bias in each independent study (e.g. study design and intervention allocation). Second, not including engineering search engines could have prevented us from finding other resources within the scope of this review. However, those missing articles published in engineer-specialized journals or conference proceedings, most likely, would have depicted the development of the device or other technical details, rather than their use for health purposes. It is most likely that experiences using drones in healthcare are published in biomedical journals, most of which are registered in the search engines herein used. Third, because of the research interest of this review, we focused on experimental studies. Given that we found a limited number of experimental studies, we recommend conducting a similar review though including descriptive literature. Fourth, we did not record reasons for exclusion while examining titles and abstracts, thus we cannot assess if we systematically excluded studies with a particular characteristic; however, it seems unlikely that this had introduced bias, because of the strict inclusion criteria we only included very specific studies (e.g. only experimental studies). Despite these limitations, this systematic review has assets too. First, to the best of our knowledge, this is the first systematic review addressing the use of drones in healthcare. Second, our search can be considered comprehensive as we included six search engines encompassing a wide range of literature.

## Conclusions

In a systematic review to assess the use of unmanned aerial vehicles (e.g. drones) in healthcare, nine studies were identified and none used these devices in real-life situations. The outcomes pursued varied from finding lost people after natural disasters to providing immediate healthcare for cardiac arrest. This review shows that research on the use of unmanned aerial vehicles for health-related purposes is limited to simulation scenarios; also, this review did not find any studies from LMICs. However, the results also show that there is a growing interest in this topic.

## Supplementary material

For supplementary material accompanying this paper visit https://doi.org/10.1017/gheg.2018.11. click here to view supplementary material
